# The Maryland Board of Dental Examiners

**Published:** 1893-04

**Authors:** 


THE MARYLAND BOARD OF DENTAL EXAMINERS, consisting of Drs. A. J. Volck, Wm. Oakley Haines and Cyrus M. Gingrich, of Baltimore, and Drs. E. S. Dashiell, of Snow Hill, and G. S. Fouke, of Westminster, met March 10th, and organized by the election of Dr. Volck, president; Dr. Dashiell, vice president, and Dr. Haines, secretary.

